# Somatic Mutation Profiles of MSI and MSS Colorectal Cancer Identified by Whole Exome Next Generation Sequencing and Bioinformatics Analysis

**DOI:** 10.1371/journal.pone.0015661

**Published:** 2010-12-22

**Authors:** Bernd Timmermann, Martin Kerick, Christina Roehr, Axel Fischer, Melanie Isau, Stefan T. Boerno, Andrea Wunderlich, Christian Barmeyer, Petra Seemann, Jana Koenig, Michael Lappe, Andreas W. Kuss, Masoud Garshasbi, Lars Bertram, Kathrin Trappe, Martin Werber, Bernhard G. Herrmann, Kurt Zatloukal, Hans Lehrach, Michal R. Schweiger

**Affiliations:** 1 Next Generation Sequencing Group, Max Planck Institute for Molecular Genetics, Berlin, Germany; 2 Department of Vertebrate Genomics, Max Planck Institute for Molecular Genetics, Berlin, Germany; 3 Department of Biology, Chemistry and Pharmacy, Free University, Berlin, Germany; 4 Department of Gastroenterology, Charité Universitätsmedizin, Berlin, Germany; 5 Berlin-Brandenburg Center for Regenerative Therapies (BCRT), Charité Universitätsmedizin, Berlin, Germany; 6 Otto Warburg Laboratory, Max Planck Institute for Molecular Genetics, Berlin, Germany; 7 Department of Human Molecular Genetics, Max Planck Institute for Molecular Genetics, Berlin, Germany; 8 Institute for Human Genetics, Ernst Moritz Arndt University, Greifswald, Germany; 9 Department of Developmental Genetics, Max Planck Institute for Molecular Genetics, Berlin, Germany; 10 Department of Pathology, Medical University, Graz, Austria; Ohio State University Medical Center, United States of America

## Abstract

**Background:**

Colorectal cancer (CRC) is with approximately 1 million cases the third most common cancer worldwide. Extensive research is ongoing to decipher the underlying genetic patterns with the hope to improve early cancer diagnosis and treatment. In this direction, the recent progress in next generation sequencing technologies has revolutionized the field of cancer genomics. However, one caveat of these studies remains the large amount of genetic variations identified and their interpretation.

**Methodology/Principal Findings:**

Here we present the first work on whole exome NGS of primary colon cancers. We performed 454 whole exome pyrosequencing of tumor as well as adjacent not affected normal colonic tissue from microsatellite stable (MSS) and microsatellite instable (MSI) colon cancer patients and identified more than 50,000 small nucleotide variations for each tissue. According to predictions based on MSS and MSI pathomechanisms we identified eight times more somatic non-synonymous variations in MSI cancers than in MSS and we were able to reproduce the result in four additional CRCs. Our bioinformatics filtering approach narrowed down the rate of most significant mutations to 359 for MSI and 45 for MSS CRCs with predicted altered protein functions. In both CRCs, MSI and MSS, we found somatic mutations in the intracellular kinase domain of bone morphogenetic protein receptor 1A, BMPR1A, a gene where so far germline mutations are associated with juvenile polyposis syndrome, and show that the mutations functionally impair the protein function.

**Conclusions/Significance:**

We conclude that with deep sequencing of tumor exomes one may be able to predict the microsatellite status of CRC and in addition identify potentially clinically relevant mutations.

## Introduction

Colorectal cancer is the third most common cancer with about 1 million cases worldwide. Over the last decades it has become clear that CRC evolves through multiple pathways and that these pathways can be roughly defined on the basis of molecular patterns such as the integrity of the mismatch repair system (MMR) or mutational and epigenetic patterns. Deficiency in the MMR is reflected in DNA microsatellite instability (MSI) which has also been associated with treatment outcome, but which needs to be further validated in additional clinical studies [1], [2], [3], [4], [5], [6].

High-throughput Sanger sequencing studies on the other hand have shown that the mutation frequency of candidate cancer genes might be much higher than expected, and that the particular combination of mutations might influence the tumor's properties [7], [8], [9], [10], [11]. With the development of next-generation sequencing (NGS) technologies the sequencing throughput has dramatically increased and the costs have decreased. In addition, and especially important for clinical settings, NGS can be applied to formalin-fixed and paraffin embedded FFPE tissue material as well as highly degraded DNA which is routinely prepared in pathology departments or found in ancient DNA [12], [13]. Several studies have used NGS technologies for the identification of the underlying mutation in monogenetic diseases [14], [15]. However, only a limited number of studies report on next-generation sequencing to identify new candidate cancer genes; one of the earliest studies examined cytogenetically normal acute myeloid leukemia, and breast cancer genomes [16], [17]. In addition, studies on malignant melanoma and small-cell lung cancer cell lines have provided first insights into genomic alterations induced by ultraviolet light exposure or tobacco smoke [18], [19].

To gain insight into the genomes of microsatellite stable and instable colorectal cancers and to identify functional relevant mutational patterns we used a hybridization based whole exome DNA capturing approach followed by 454 next generation sequencing [20]. Applying stringent bioinformatics analyses, we narrowed down the amount of functionally significant somatic mutations in MSI to 359 and 45 in MSS cancers, thus highlighting specific mutation patterns depending on the microsatellite status. We were able to confirm our results by sequencing the exomes of four additional CRC cases (one MSI, three MSS) using a different enrichment and sequencing technology. Among these mutations are BRAF in the MSI cancer and KRAS and TP53 in the MSS cancer, further underscoring the validity of our selection approach [21]. Further functional characterizations identified recurrent somatic mutations in BMPR1A, a protein which has been associated so far with juvenile polyposis syndrome, a cancer predisposition syndrome.

## Results

### Sequence-specific enrichment and sequencing strategy

We sequenced tumor and matching normal colon tissues from two patients with high grade adenocarcinoma of the colon (G3), patient 1 with a microsatellite instable and patient 2 with a microsatellite stable tumor (Table 1, Figure S1). For the determination of germline mutations we sequenced in addition to the tumor tissues from each patient adjacent not affected normal colonic tissue. Using Illumina sequencing and SNP arrays we determined that the tumor of patient 1 is copy number stable whereas patient 2 showed variations which we used for the re-evaluation of identified high stringency mutations (Table S3).

**Table 1:** Colorectal cancer patients selected for NGS.

	Patient 1	Patient 2
**Age**	59	65
**Gender**	male	Male
**Grade**	G-3	G-3
**Localization**	proximal CRC	proximal CRC
**MS status**	MSI	MSS
**CNV**	no	Yes

**Figure 1:**
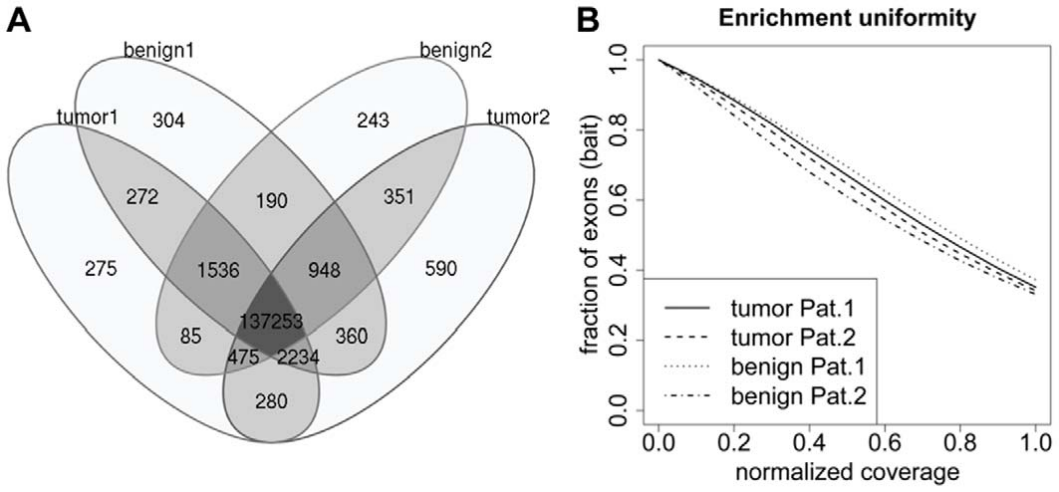
Qualities of the targeted whole exome sequencing approach. (A) Venn diagram of captured exons of normal and tumor samples. Captured exons with at least one read were counted. (B) Representative normalized coverage-distribution plot. The fraction of bait-covered exons in the genome achieving coverages equal or lower than the normalized coverage is indicated on the x-axis. The mean coverage per exon was divided by the mean coverage of all exons.

We analyzed the complete exomes of more than 135,000 exons with single-read shotgun 454 sequencing (Figure 1, Figure S1, Figure S2, Table 2). To assess the effect of coverage depth on the sensitivity and specificity of sequence variant detection, genotype calls of the Affymetrix SNP array 6.0 were compared step-wise to the called nucleic acid positions and resulted in an accuracy of more than 99% (Figure S3). In addition to the SNP array, we used Sanger sequencing to confirm 23 selected mutations (Table S5, Figure S5).

**Table 2:** Tumor and normal genome coverages from MSI and MSS cancer patients.

	Patient 1	Patient 2
	normal tissue	tumor tissue	normal tissue	tumor tissue
mapped reads (% of all reads)	5,659,707 (99.67%)	5,569,487 (97.21%)	2,425,905 (97.17%)	4,624,656 (96.71%)
unique mapped reads (% of all reads)	5,180,233 (91.23%)	5,285,822 (92.26%)	2,304,598 (92.38%)	4,367,855 (91.34%)
unique mapped bases (bp) (% of all bases)	1,978,702,340 (92.18%)	2,045,499,143 (88.22%)	883,388,420 (94.62%)	1,916,322,803 (94.53%)
median read length (bp)	393	418	418	483
unique reads in target region (% of all reads)	4,501,660 (79.28%)	4,477,985 (78.16%)	1,919,239 (67.88%)	3,640,778 (76.14%)
Target Base Coverage (%)	95.58	94.82	93.79	94.96
regions hit (of 176,159)	150,763	149,121	142,982	143,424

### Identification of somatic mutations in coding sequences for a MSI CRC

**Figure 2:**
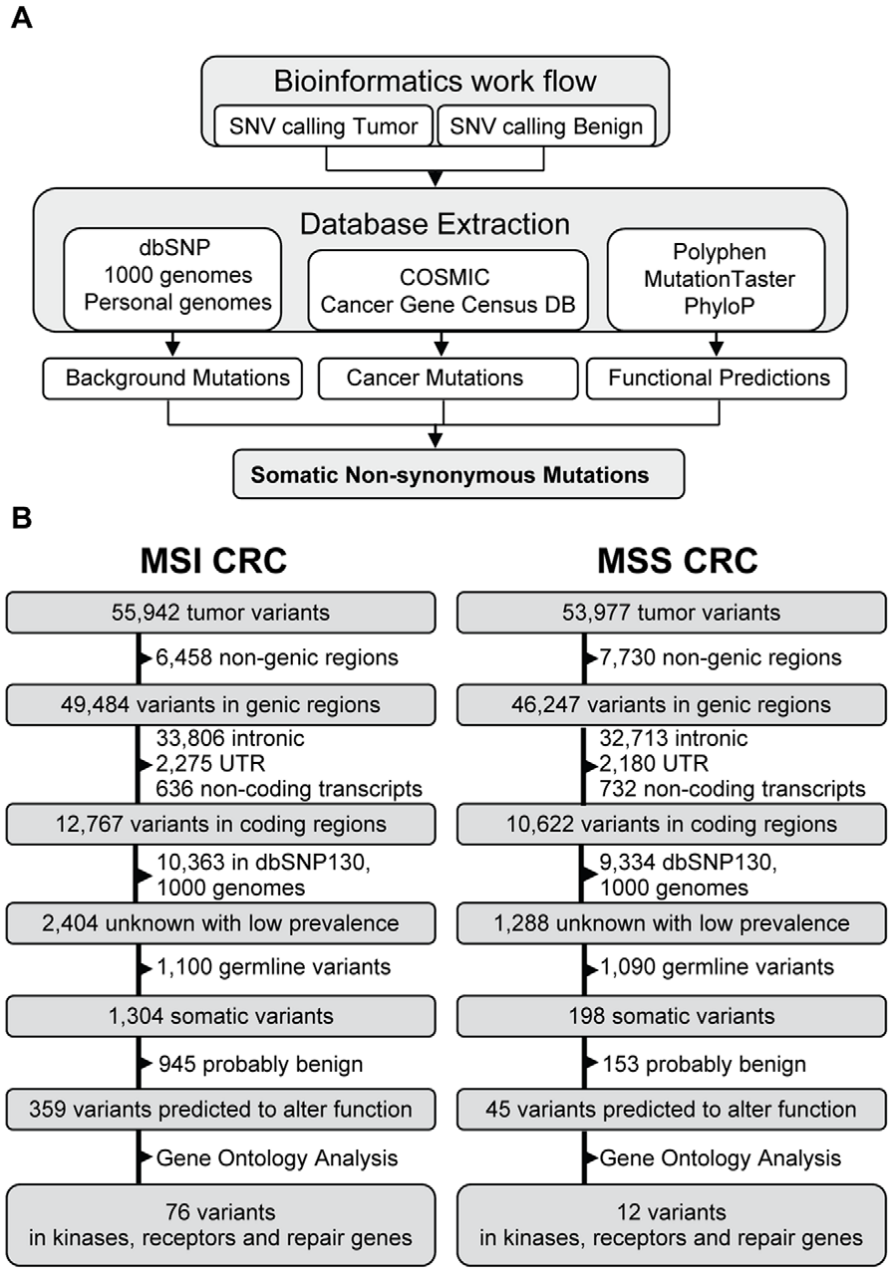
Identification process of somatic relevant SNVs. (A) Schematic of the bioinformatics SNV detection workflow. (B) Extraction of functionally relevant somatic mutations for MSI and MSS colorectal cancers. Variants were detected with the GS Reference Mapper before they were filtered for their localization, annotation in dbSNP130 or the 1000genomes, somatic and functionally impairment. From dbSNP130 or the 1000genomes variants with frequencies above 1% were used. For MSI CRC 359 variants and for MSS CRC 45 with predicted altered protein functions were identified.

Searching for variants in coding regions we found 12,767 and 12,518 small nuclear variations in 6,428 and 6,205 genes, for tumor and normal respectively. Of these variants 1,428 for control and 2,404 for tumor have an average heterozygosity or minor allele frequency lower than 1% or have not been previously reported in dbSNP or the 1000 Genomes Project (Figure 2). Since indels (small insertions and deletions) at homopolymeric sites are a major source of sequencing errors of the 454 platform we ignored this type of alteration in our analyses.

Our somatic variant detection strategy was designed to minimize false positive somatic variant calls rather than to determine zygosity. We used a two-step approach with two different stringency levels to detect variants in tumor and benign tissues similar to Pleasance *et al.* [18]. In the first step, tumor variants were called under stringent criteria, which were determined by comparison to the SNP genotyping array data. The second step ascertained whether the tumor variant was germline or somatic. To keep the false negative rate in the benign tissue at less than 10%, we set the coverage cut-off at 5-fold, below which no conclusions were drawn regarding whether a variant was somatic or germline. If the coverage cut-off was met, a single read showing the same variant in benign and tumor tissue resulted in the categorization of the variant as germline.

Using this strategy we identified 915 somatic non-synonymous mutations affecting 864 genes. The majority of somatic mutations were missense mutations (65%). However, many (7%) are located within untranslated regions of genes and might therefore result in altered expression or increased decay of mRNA species. Furthermore, approximately 0.5% of these mutations are found at splice sites and could influence splicing events, leading to an altered transcriptome structure. In addition, three somatic variants were identified in miRNA regions (Table S6). These mutations are of particular interest because miRNAs have been implicated as master regulators of tumor homeostasis. Analysis of the specific types of nucleic acid variations, including known and unknown variants, showed essentially the expected rates of nucleotide exchanges, as determined by calculations using dbSNP130 (Figure S4).

### Functional analysis of mutations for a MSI colon cancer

Since not all of the 1,304 somatic mutations are likely to be pathologically relevant, we sought to identify those that probably destroy protein function or affect highly conserved amino acids and might therefore be functionally important. We used Polyphen and MutationTaster classification tools to predict the functional consequences of amino acid changes or frameshift mutations and found that 359 genes had at least one potentially destructive mutation (Table S1) [22], [23].

Of the potentially destructive somatic mutations, 309 were located in positions highly conserved in 44 different species, including opossum *(Monodelphis domestica)*, chicken *(Gallus gallus)* and lamprey *(Petromyzon marinus)*. Of these, 259 were located in genes expressed in the colon, of which 47 were repair, receptor, or kinase genes. Visualization of selected mutations on protein structures indicates that these nucleotides are on the protein surface, potentially resulting in disrupted protein-protein interactions.

The Catalog of Somatic Mutations in Cancer (COSMIC) database is a comprehensive collection of cancer-related mutations. Approximately 39% of our mutated genes were already described in this database, and 13% were found by Wood *et al* [10]. As did these previous databases, we found the *BRAF* p.V600E mutation in the MSI case and we identified *KRAS* and *TP53* mutations in the MSS tumor. *BRAF* mutations are found in approximately 10% of CRCs, predominantly MSI and 30 to 35% of all patients with sporadic colorectal cancers carry somatic *KRAS* and *TP53* mutations. These findings further demonstrate the sensitivity of our classification strategy.

### Mutational landscape of a MSS colon cancer

For the MSS colon cancer we identified 10,622 small nuclear variations. After the same filtering processes as for the MSI cancer using the dbSNP database and the data from the 1000 Genomes Project 1,288 variants remained which either had low prevalence or were unknown. Of these, 198 were somatic and 45 were predicted to alter gene function based on MutationTaster and Polyphen calculations (Figure 2B, Table S2) [22], [23]. In regard to copy number variations five of the 45 identified mutations are located within amplified regions, and, as expected, none in regions with deletions. The ratios of reads with reference sequence to mutated sequence are not exceeding ratios in copy number stable areas which supports the SNV-calling algorithm.

In contrast to 1,304 somatic mutations in the MSI tumor we found 198 somatic mutations in the MSS tumor which demonstrates that the defective MMR system in MSI tumors results in a significant increase in mutation rates in colorectal cancer.

**Figure 3:**
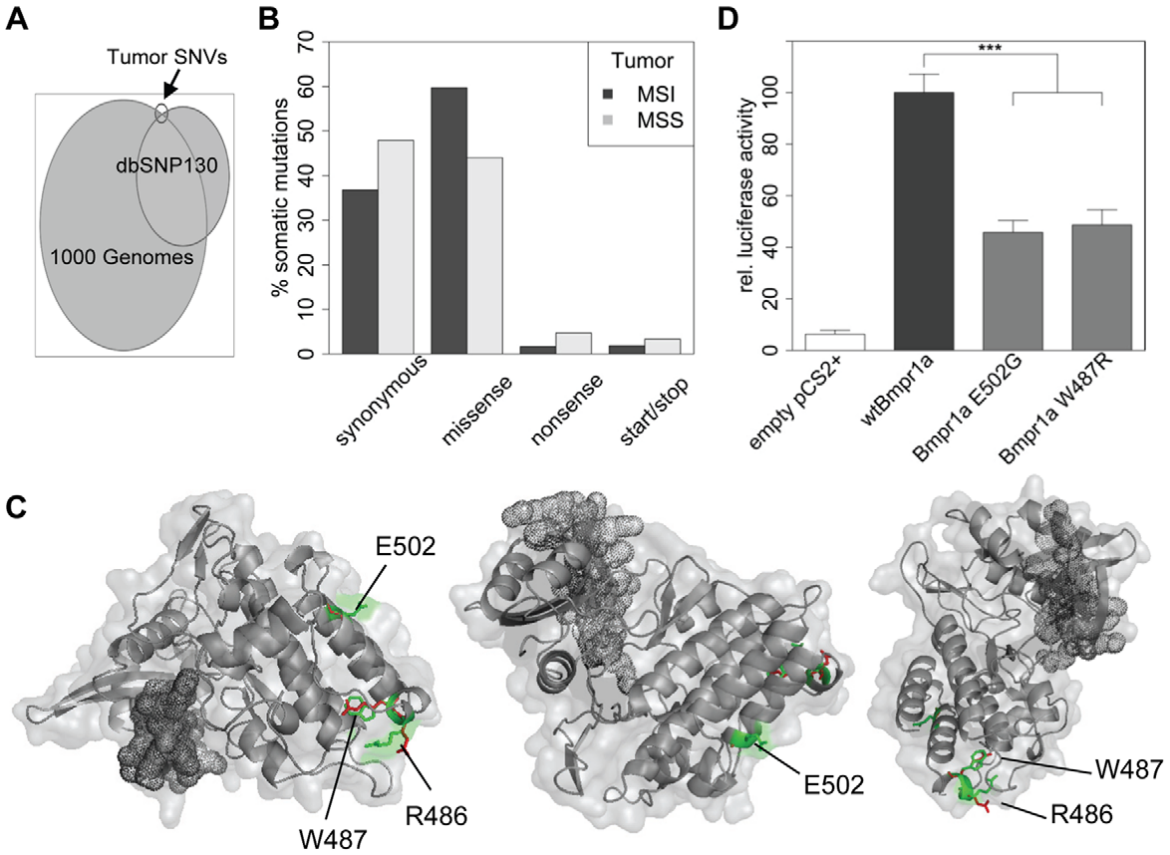
Characterization of primary identified SNVs. (A) Proportional Venn diagram. Fractions of called SNVs identical to the Genomes Project data and dbSNP130. Only data for which the minor allele frequency or the average heterozygosity was known and below 1% were used for comparison. (B) Distribution of synonymous, missense, nonsense and mutations affecting the start or stop codon are shown in relation to all somatic mutations. (C) BMPR1A mutations p.W487R and p.E502G are located at the protein kinase domain of BMPR1A. Reference amino acids are in green, the mutated forms are shown in red. The net structure at the left lower side indicates the ATP binding domain. (D) BMPR1A mutations show decreased signaling acitivity. Activity of wt mBMPR1A, mBMPR1A E502G and mBMPR1A W487R was determined in C2C12 cells using a SMAD-responsive Luciferase reporter gene assay. Induced Luciferase activity was normalized to Renilla acitivty. The activity of untransfected cells was set to 0% and the activity of wt mBmpr1a was set to 100%. Significant differences were calculated with a two-tailed t-test and marked as: * p≤0.05, ** p≤0.01, *** p≤0.001.

Furthermore, looking at intersections between both cancer types we found BMPR1A, WDTC1 (WD and tetratricopeptode repeats 1) and EHD3 (EH-domain containing 3) mutated in both tumors. The selection was based on functional impairment with high probability in Polyphen and MutationTaster [22], [23]. All mutations are located on the surface of the protein and are highly conserved. Since we found significant cancer-related pathways associated only with BMPR1A but not with WDTC1 or EHD3, and in addition germline mutations in BMPR1A are a known risk factor for juvenile polyposis syndrome, we chose BMPR1A for additional functional assays (Figure 3, Figure S5). Using reporter assays with wild type and mutated BMPR1A proteins we were able to show that the mutated proteins are strongly impaired in their signalling function and that stimulation with BMP2 results in a reduced maximum activity (Figure 3D).

### MSI colorectal cancers harbour up to 8-fold more coding somatic mutations than MSS cancers

The analyses presented so far have been based on 454 whole exome sequencings of one colorectal cancer patient for each microsatellite status. To further confirm that the increased amount of coding mutations in MSI cancers can be generalized and is not due to the technology used (‘array’ enrichment and 454 sequencing) we sequenced the exomes of four additional colorectal cancers, each with matching normal tissues. This time we used ‘in solution hybridization’ for capturing of DNA followed by SOLiD sequencing. After the same filtering procedures as described for the first two patients we again determined up to 8-fold higher mutation rates for the MSI colorectal cancer than for MSS cancers (Table 3). In this regard we found 532 non-synonymous somatic SNVs in the additional MSI CRC and only 65, 74 and 76 in the three MSS CRC cases. Thus, the differences in mutation rates are reproducible and independent of the sequencing technology used.

**Table 3:** Distribution of SNVs in MSI and MSS tumors.

	Patient 1	Patient 2	Patient 3	Patient 4	Patient 5	Patient 6
**MS status**	MSI	MSS	MSS	MSS	MSS	MSI
**enrichment technology**	array	array	in solution	In solution	in solution	in solution
**NGS technology**	454	454	SOLiD	SOLiD	SOLiD	SOLiD
**number of mutations** *	897	124	65	74	76	532

*non-synonymous somatic mutations, not annotated in dbSNP.

## Discussion

Using next-generation sequencing, we sequenced the exomes of MSS and MSI colon cancer patients, with mean coverages of approximately 20-fold. We applied a two-sided classification algorithm to uncover functionally relevant mutations. Using this approach, we demonstrate for the first time that an array-capture NGS one-step work flow is a powerful tool for deep characterization of solid tumors and show that MSI tumors carry eight times more functional relevant mutations than MSS tumors (Figure 2).

The functional impact of the somatic variations was predicted using two functional prediction algorithms, Polyphen and MutationTaster, and we found 359 somatic mutations for the MSI and 45 for the MSS cancer that are highly likely to cause functional impairment [22], [23]. The heterogeneity of mutated genes suggests that not a specific gene *per se* but the affected pathway plays a major role for tumor development. In this regard we find a significant enrichment of mutations in cancer-related pathways such as cancer, cellular development and DNA replication, recombination and repair (Table S5). Interestingly, we find 50% of the most significant enriched pathways in the MSS cancer also as significantly enriched pathways for the MSI cancer, indicating that even though MSI cancers harbour an increased number of mutations both cancers might develop through overlapping pathomechanisms.

Historically, microsatellite testing in colorectal cancers was the first predictive test for the identification of an underlying mismatch repair (MMR) mutation. Since more than 90% of hereditary non-polyposis colorectal cancers (HNPCC) show MSI, the microsatellite status has become a common diagnostic marker of MMR. In addition, survival advantages and therapeutic consequences have been reported for patients with MSI tumors [5], [24]. Using ultradeep sequencing of one conserved region, UCR41, de Grassi and colleagues show that this region has higher mutation rates in HNPCC samples than in healthy controls and suggest that this might be used as sensitive molecular assay of genomic instability [24]. We have extended their analyses to whole exomes and found 8-fold differences in the numbers of somatic mutation of MSI and MSS colorectal cancers. In comparison, a study by Greenman *et al.* which reported on the sequencing of 518 protein kinase genes in 210 diverse human cancers found an approximately 25-fold higher mutation rate for MMR-deficient cancers [9]. However, these are extrapolations and in contrast to our study they included tumors from different origins and examined all somatic mutations irrespective of their functional relevance. With our sequencing approach we also detected a somatic *MLH3* mutation in the MSI tumor, which, even though *MLH3* mutations do not belong to the classical MMR mutations in CRC, might contribute to the microsatellite instability phenotype [25]. Furthermore, the combination of MSI and *BRAF* mutation, as detected for the MSI tumor described, is most frequently found for CpG island methylation phenotype 1 (CIMP1) tumors which are associated with *MLH1* promoter methylations [21], [26]. The promoter methylation in turn is associated with gene silencing mechanisms which is suggestive as an explanation for the MSI status of the tumors. On the other side, it has been proposed that chromosomal instability (CIN) and CIMP represent two independent and inversely related mechanisms of instability [27]. CIMP-negative cases are associated with p53 (71%) and *KRAS* (33%) mutations, but are rarely found with *BRAF* (2%) mutations. Since we found large copy number variations as well as *KRAS* and *TP53* mutations in the MSS tumor analyzed this tumor is most likely CIMP-negative [5], [24].

The sequencing and analysis strategy we have presented might be the basis for future classification tools for colorectal cancers because it may allow a parallel detection of an increased mutation frequency in MSI tumors as well as the detection of the underlying MMR defect. In addition, we were able to detect mutations of genes frequently associated with certain subtypes of colorectal cancers such as *BRAF, KRAS* and *TP53*. Within our high priority genes, encompassing all genes which pass all selection filters, *BMPR1A* stands out as mutated in both cases. The overall structure reveals that the mutated amino acids are all located at the C-terminal intracellular helix bundle at the protein kinase domain and suggests that protein-protein interactions are destroyed [28]. Germline *BMPR1A* mutations predispose to juvenile polyposis syndrome; however, our findings indicate that also somatic mutations might play an important role in sporadic colorectal cancer development [29], [30], [31], [32], [33].

Besides these mutations we have also identified several mutated cancer drug targets or genes that are associated with treatment outcome, including *BRAF, KRAS, FGFR2* and *MTOR*, which might help to choose optimal drug combinations. As such similar targeted re-sequencing approaches and bioinformatics filtering strategies might become a gold standard for individually tailored colorectal cancer treatment in the future.

## Materials and Methods

### Ethics Statement

The study has been approved by the Ethical Committee of the Medical University of Graz. For new samples patients have given their written informed consent. For old samples (15 years old) no informed consent was available, therefore all samples and medical data used in this study have been irreversibly anonymized.

### Case presentation and tissue sample collection

Patient 1 had a high-grade (G3) adenocarcinoma of the proximal colon, staged pT-3C, pN-0, pM-X, pR-0, microsatellite instable (Figure 1A). In addition, this case was selected because of its chromosomal stability, as determined using genome-wide next generation sequencing (NGS) (Figure 1B). Patient 2 had a high-grade (G3) adenocarcinoma of the proximal colon, staged pT-4B, pN-2, pM-X, microsatellite stable.

Human tissue obtained during surgery was snap-frozen in liquid nitrogen. Cryosections (3 µm thick) were prepared and stained with haematoxylin and eosin to evaluate tumor cell content. Dissections were performed under the microscope to achieve a tumor cell content of >80%. DNA isolation was performed using the QIAamp DNA Mini Kit (Qiagen), according to the manufacturer's instructions.

### Whole Exome DNA Enrichment and Genome Sequencer FLX sequencing

Genomic DNA of both tissues was subjected to whole exome sequence capture using Roche/NimbleGen's 2.1M Human Exome Array. This array is based on build 36.3 of the human genome sequence, and captures the coding regions of 16,755 NCBI RefSeq genes (approximately 180,000 coding exons) as well as 493 miRNA regions. Tumor and normal tissue DNA were subjected to whole exome sequence capturing according to the manufacturer's protocol. DNA was sheared by nebulization to fragment sizes below 800bp, cleaned (Zymo Research) and end-polished using T4 DNA Polymerase and T4 Polynucleotide Kinase. Linker adapters pSel3 (5′ – CTCGAG AAT TCT GGA TCC TC – 3′) and pSel4-P (5′ – Phos/GAG GAT CCA GAA TTC TCG AGT T – 3′) were ligated and size selection was performed using AMPure DNA Purification Beads (Agencourt). Quality was controlled with the Bioanalyzer system. LM-PCR was performed with LMPCR3 primers (5′ – CUC GAG AAU UCU GGA UCC UC – 3′) before the library was used for hybridization at 42°C for 72h. The arrays were washed two times at 47.5°C, two times at room temperature and two times at 42°C with washing buffers as recommended. Bound genomic DNA was eluted with 125 mM NaOH for 10 min at room temperature and amplified by LM-PCR using primers LMPCR3. Captured amplified samples were subjected to quantitative PCR to measure the relative enrichment.

The enriched Nimblegen DNA was used to construct single-stranded Genome Sequencer FLX (454/Roche) libraries. After emulsion PCRs sequencing primers were annealed to the template and beads were incubated with Bst DNA polymerase, apyrase, and single-stranded binding protein. Pyrosequencing was performed on a 70×75 mm picotiter plate in 13 separate sequencing runs. After default raw data processing, a resequencing trimming filter was used to increase the data output. (Parameters used: doValleyFilterTrimBack  =  false, vfBadFlowThreshold  = 6, vfLastFlowToTest  = 168, errorQscoreWindowTrim  = 0.01).

For the sequencing we performed 13 Genome Sequencer FLX runs, which produced over 558 million bases and 1.43 million reads per run. Reads were aligned to the human reference genome, NCBI build 36 (http://hgdownload.cse.ucsc.edu/goldenPath/hg18/), using GS Reference Mapper Version 2.0.0.12 (Roche). The best matches in the genome were used as the location for the reads with multiple matches. Only unique reads with a minimum length of 50 bp were used for further analysis (see run statistics Tab. 2).

Detection of variants was performed with the GS Reference Mapper Version 2.0.0.12 (Roche). Redundant reads were subtracted before variant callings. Only the HCDiff (high confidence differences) of the GS Mapper software were used as basis of variant detection [20]. HCDiff callings presume at least three reads with the variant with both forward and reverse reads included; alternatively the quality scores at the variable positions must be over 20 (or over 30 if a homopolymer of five or more bases is involved). As additional quality criteria we used only variants with a coverage >10× of high quality reads.

### Whole Exome ‘in solution’ DNA Enrichment and SOLiD sequencing

Enrichments and SOLiD library preparation were performed according to Agilent's SureSelect Target Enrichment protocol for the Applied Biosystems SOLiD system. In brief, whole genomic DNA was sheared and end repaired. For adapter ligations 30x excess of the adapters were used. Size selections for 150–200 bp DNA fragments were performed followed by a nick-translation and amplification step with Platinum polymerase (Invitrogen) and Pfu-Polymerase (Fermentas). For hybrid selection the libraries were adjusted to 500 ng in 3.4 µl volume and added to the SureSelect Block solutions. Hybridizations were performed for 24 h at 65°C, hybrids were extracted with 500 ng M-280 streptavidin Dynabeads (Invitrogen) and finally eluted with 50 µl Elution buffer. After amplification with Platinum polymerase the libraries were quantified by qPCR and DNA concentration was titrated to achieve a fraction of 10–20% monoclonal template beads in the emulsion PCR using in total 0.7 to 1 billion beads. Successive bead enrichment and deposition of 130 million beads per quarter slide (quad) was followed by standard 50 bp fragment runs. Each of the four patient samples was analyzed on a single quad.

Mapping was performed with the Bioscope alignment pipeline using the seed & extend algorithm with a mismatch penalty score −2.0. Single Nucleotide Variants (SNV) were called with the DiBayes algorithm integrated in the Bioscope package.

### Single nucleotide variant (SNV) detection

Tissue materials were genotyped on the Affymetrix 6.0 array, according to the manufacturer's protocol. Array positions with a quality score (p-value) <0.1 were used for comparison with the sequencing data. Sequencing data positions were used if their coverage exceeded 3-fold. This generated 46,000 and 49,000 positions for tumor and benign tissue, respectively, that were eligible for comparison. To determine false positive and false negative rates, we set the array data as standard and distinguished between reference call and SNP call dependence on the array data.

For the detection of somatic variants, a bimodal strategy was applied with tumor variants called under stringent criteria, whereas variants in control tissue were called using less stringent criteria: A minimum threshold for reads was set with variants of 15% of all reads at a given position in tumor. Less stringent criteria were used for calling control tissue variants with a minimum of one variant read and a minimum coverage cutoff of 5. For coverages above 30-fold one variant read was accepted.

### Capillary Sequencing

Follow-up confirmation of identified SNVs was performed on an ABI 3730 (Applied Biosystems) capillary sequencing instrument following standard procedures.

### Determination of Copy number variations

Preparation of single read libraries and sequencing were performed using the Solexa sequencing platform (GenomeAnalyzer IIx, Illumina) following the manufacturer's instructions. Image Analysis and base calling were performed using Firecrest 1.9.5_14 and Bustard 1.9.5_14 and reads were aligned to the human genome (NCBI36) using Bowtie 0.9.7.1 [34]. Copy number analysis was done in R using the DNAcopy package [35]. In short, DNA read frequencies were determined for bins of 50 Kb. The log2 frequency ratio of corresponding bins was calculated for tumor versus normal tissue. Median of ratios was centered to zero experiment wise. Log ratios were smoothed by DNAcopy using default values and copy number variation was detected by DNAcopy using a threshold of two standard deviations.

Genotyping on the Affymetrix 6.0 array was performed according to the manufacturer's protocol. Regions of copy number gain and loss were determined by paired and analysis using the Hidden Markov Model (HMM) of the Partek Genomics Suite software (Partek Inc, St.Louis, MO) with default parameter settings. For paired analysis, copy number values were generated by comparing tumor and benign tissue profiles from the same patient.

### Bioinformatics workflow

For each tissue, variations were annotated using the gene models generated by Ensembl (ensembl 54.36, www.ensembl.org). All variations were mapped to all transcript models, which led to multiple annotations for several loci. For instance, a variant can lead to an amino acid change in one transcript and appear in the UTR of another. Comparison of tumor and benign tissue variants to dbSNP130 and 1000 Genomes Project data was carried out with the subset of dbSNP130 and 1000 Genomes Project positions with minor allele frequencies or average heterozygosity >0.01. Variants were subjected to many comparisons with external data sources. Most data sources are integrated in the UCSC genome browser (http://hgdownload.cse.ucsc.edu/goldenPath/hg18/database/) or were derived from websites like the 1000 Genomes Project (ftp://ftp.1000genomes.ebi.ac.uk/vol1/ftp/release/2009_04/) the gene ontology data (http://archive.geneontology.org/full/2009-10-01/go_200910-termdb.obo-xml.gz), the cosmic database version 46 (http://www.sanger.ac.uk/genetics/CGP/cosmic/) or the cancer gene census database (http://www.sanger.ac.uk/genetics/CGP/census/). Functional classifications were performed using Polyphen and MutationTaster classification tools (http://genetics.bwh.harvard.edu/pph/, http://neurocore.charite.de/MutationTaster/) [22], [23]. Base conservation among 44 species was tested using the phyloP track of the UCSC browser (http://genome.cshlp.org/content/early/2009/10/26/gr.097857.109.abstract). Bases were considered highly conserved if their conservation score was greater or equal 2.0 (0.975 quantil of all conservation scores). For gene expression healthy control samples from http://www.ncbi.nlm.nih.gov/sites/GDSbrowser?acc=GDS2609 were used. We calculated genewise mean expression values across all samples and used the first quartile as threshold to determine gene expression. Pathway analyses were performed with the ingenuity pathway analysis tool (http://www.ingenuity.com). All new data from this study has been deposited at NCBI dbSNP database of genetic variation (user-name MPIMGCancerogenomics). Accession numbers are included as Table S4.

### Protein structure modeling

Models for BMPR1A were obtained from SwissModel and ModBase. Very similar models except for some loops (total Calpha rmsd 0.66) were rendered in PyMol [36], [37].

### Reporter assays

The activity of the wildtype mouse protein (wtmBMPR1A) and its mutants was determined by measuring induced Luciferase activity in the transiently transfected pre-myoblastic mouse cell line C2C12 (ATCC). WtmBMPR1A was amplified from mouse cDNA and cloned in the expression vector pCS2+. Both mutations W487R and E502G were inserted by Quikchange mutagenesis (Stratagene) using the following primer pairs:

mBmpr1a_W487R_fwd caatcgtgtctaaccgcCggaacagcgatgaatg;

mBmpr1a_W487R_rev cattcatcgctgttccGgcggttagacacgattg and

mBmpr1a_E502G_fwd gttttgaagctaatgtcagGatgttgggcccataatc;

mBmpr1a_E502G_rev gattatgggcccaacatCctgacattagcttcaaaac.

C2C12 cells were cultured in DMEM glucose 4,5 g/L with 10% FCS were co-transfected with each Bmpr1a expression construct, a Smad Binding Element (SBE) luciferase construct [38] and the normalization vector pRL-Tk (Promega Corporation, Madison, WI, USA) using Turbofect (Fermentas GmbH, St. Leon-Rot, Germany). Luciferase activity was determined as described previously [39].

## Supporting Information

Figure S1 Quality controls of the colon cancer case 1 and experimental performances. (A) Visualization of Copy number variations (CNV) using Illumina sequencing for MSI and MSS cancers. Chromosomal coverage ratio of tumor versus benign tissue sample. Each chromosome was divided into 50-kb bins. The log2 ratios of unique reads per bin are plotted across all chromosomes. The red lines depict the local averages as calculated by DNAcopy [35]. (B) Influence of sequencing depth on exon capture coverage (left) and SNV detection (right). Exon coverage and SNVs in the enrichment regions were determined after each sequencing run. The numbers of exons covered and the number of SNVs detected at different coverage levels were compared for tumor and benign tissue separately. Sigmoid functions Y = c+(d-c)/(1+exp(b)*(log(X)-log(e)) were used to fit the data and extrapolate the saturation level.
(TIF)

Figure S2 Sequence coverage along a contiguous target. (A) The base-by-base sequence coverage along a typical 80-kb segment (*BRCA1* gene) in the UCSC browser is shown. The 10-fold coverage level is highlighted by a black line. (B) Coverage profiles of exon targets depending on exon size. Exons have been divided into four groups depending on exon size. Coverages were calculated in relation to the relative position on the exon and averaged by the mean over all exons of the group.
(TIF)

Figure S3 Comparison between the SNP array and NGS. About one million known SNP positions have been investigated using the Affymetrix human whole genome SNP array 6.0. Array positions with a quality score (p-value)<0.1 and sequencing positions with coverage exceeding 3-fold coverage were used for comparison. Forty thousand and thirty-six thousand positions for tumor and benign tissue, respectively, were eligible for comparison. To determine false positive and false negative rates, the array data was set as standard and between reference call and SNP call dependence on the array data was distinguished. (A) homo- and heterozygous SNVs were discerned (B) for the calculation of the haploid concordances heterozygous positions were counted as homozygous non-reference positions.
(TIF)

Figure S4 Nucleotide exchange rates in DNA from tumor and benign tissue, as compared to dbSNP130 data for patient 1. (A) Using the GS Reference Mapper Version 2.0.0.12 (Roche), software nucleotide exchanges were calculated for all possible transitions (e.g. A<>G, A<>C). Dark grey: tumor, grey: benign, light grey: dbSNP (B) Dinucleotide context for single nucleotide variants from tumor and benign tissue. Dark grey: tumor, grey: benign, light grey: dbSNP
(TIF)

Figure S5 Validation, visualization and pathway analyses. (A) Visualization of the Sanger and 454 next generation sequencing result of BMPR1A. Red arrows indicate the location of the mutation. (B) Ingenuity pathway analysis of BMPR1A, WDTC1, EHD3 and CTR9 (top) and visualization of the conservation of BMPR1A p.W487 and p.E502 across human, mouse, chicken, zebrafish and other organisms (bottom).
(TIF)

Table S1 List of 359 somatic candidate genes with functionally relevant mutations for the MSI colorectal cancer case. Column headings are as follows: (A) Location, (B) Mutation, (C) coverage in tumor, (D) number of reads with SNV in tumor, (E) coverage in normal, (F) number of reads with SNV in normal, (G) amino acid position, (H) amino acid, (I) mutated amino acid, (J) gene name, (K-M) Ensembl transcript ID, Ensembl gene ID, Ensembl protein ID, (N) nucleotide conservation (PhyloP), (O) protein domain, (P) Polyphen, (Q) MutationTaster, (R) mean expression in colon, (S) described in Wood et al.2007, (T) listed in COSMIC database, (U) listed as CancerGeneCensus (dom = dominant (oncogene), rec = recessive (tumor suppressor)), (V-Y) listed in GO database as repair gene, kinase, receptor, transmembrane receptor, (Z-AJ): annotated within dbSNP130 (rs numbers indicate mutations with frequencies above 1%), 1000 genomes, Venter genome, Watson genome, Yoruban genome, Corean genome, Han genome, genome 12891, genome 12878, genome 12892, genome 19240.
(XLS)

Table S2 List of 45 somatic candidate genes with functionally relevant mutations for the MSS colorectal cancer case. Column headings are as follows: (A) Location, (B) Mutation, (C) coverage in tumor, (D) number of reads with SNV in tumor, (E) coverage in normal, (F) number of reads with SNV in normal, (G) amino acid position, (H) amino acid, (I) mutated amino acid, (J) gene name, (K-M) Ensembl transcript ID, Ensembl gene ID, Ensembl protein ID, (N) nucleotide conservation (PhyloP), (O) protein domain, (P) Polyphen, (Q) MutationTaster, (R) mean expression in colon, (S) described in Wood et al.2007, (T) listed in COSMIC database, (U) listed as CancerGeneCensus (dom = dominant (oncogene), rec = recessive (tumor suppressor)), (V-Y) listed in GO database as repair gene, kinase, receptor, transmembrane receptor, (Z-AJ): annotated within dbSNP130 (rs numbers indicate mutations with frequencies above 1%), 1000 genomes, Venter genome, Watson genome, Yoruban genome, Corean genome, Han genome, genome 12891, genome 12878, genome 12892, genome 19240.
(XLS)

Table S3 Copy number variations (CNVs) of the MSS colon cancer case. Somatic amplifications and deletions were determined with the Affymetrix 6.0 array followed by a paired Hidden Markov Model analysis with the Partek genomics Suite software. Column headings are as follows: Chromosome, start and end position of the CNV, cytoband, copy number, length in bp and CNV state.
(XLS)

Table S4 Ingenuity pathway analysis of the MSI and MSS CRC. Selected were pathways listed in the top 25 significantly enriched pathways for the MSS cancer which were also found to be highly significant in the MSS cancer.
(XLS)

Table S5 List of candidate locations used for capillary sequencing. Column headings are as follows: (A) Chromosomal localization, (B) validation with Sanger capillary sequencing, (C) reference codon, (D) position of the mutation in the reference codon, (E) mutated codon, (F) amino acid position, (G) total amount of amino acids, (H) reference amino acid, (I) mutated amino acid, (J) protein domain, (K-M) Ensembl transcript ID, Ensembl gene ID, Ensembl protein ID, (N) gene name, (O) listed in COSMIC database, (P) described in Wood et al.2007, (Q-AA): annotated within 1000 genomes, dbSNP130, Venter genome, Watson genome, Yoruban genome, Corean genome, Han genome, genome 12891, genome 12878, genome 12892, genome 19240, (AB) number of reference reads in tumor, (AC) number of mutated reads in tumor, (AD) number of reference reads in normal, (AE) number of mutated reads in normal, (AF) MutationTaster, (AG) Polyphen
(XLS)

Table S6 SNVs in miRNA regions. Column headings are as follows: (A) Patient ID, (B,C) chromosomal localization, (D) mirBase13 including the ID of the mutated miRNA, (E) Percentage of reads with the mutation in the tumor tissue, (F) Percentage of reads with the mutation in normal tissue, (G) annotation of the SNV, (H) related gene name, (I) mutated nucleotide, (J) annotation in dbSNP 130, (K) annotation in the 1000genomes project
(XLS)

Table S7 NCBI numbers of the identified SNVs.
(XLS)
