# Supplementary figures and images for: Somatic Mutation Profiles of MSI and MSS Colorectal Cancer Identified by Whole Exome Next Generation Sequencing and Bioinformatics Analysis
Source: PLoS One. 2010 Dec 22;5(12):e15661. doi: 10.1371/journal.pone.0015661 (PMC3008745; doi:10.1371/journal.pone.0015661)

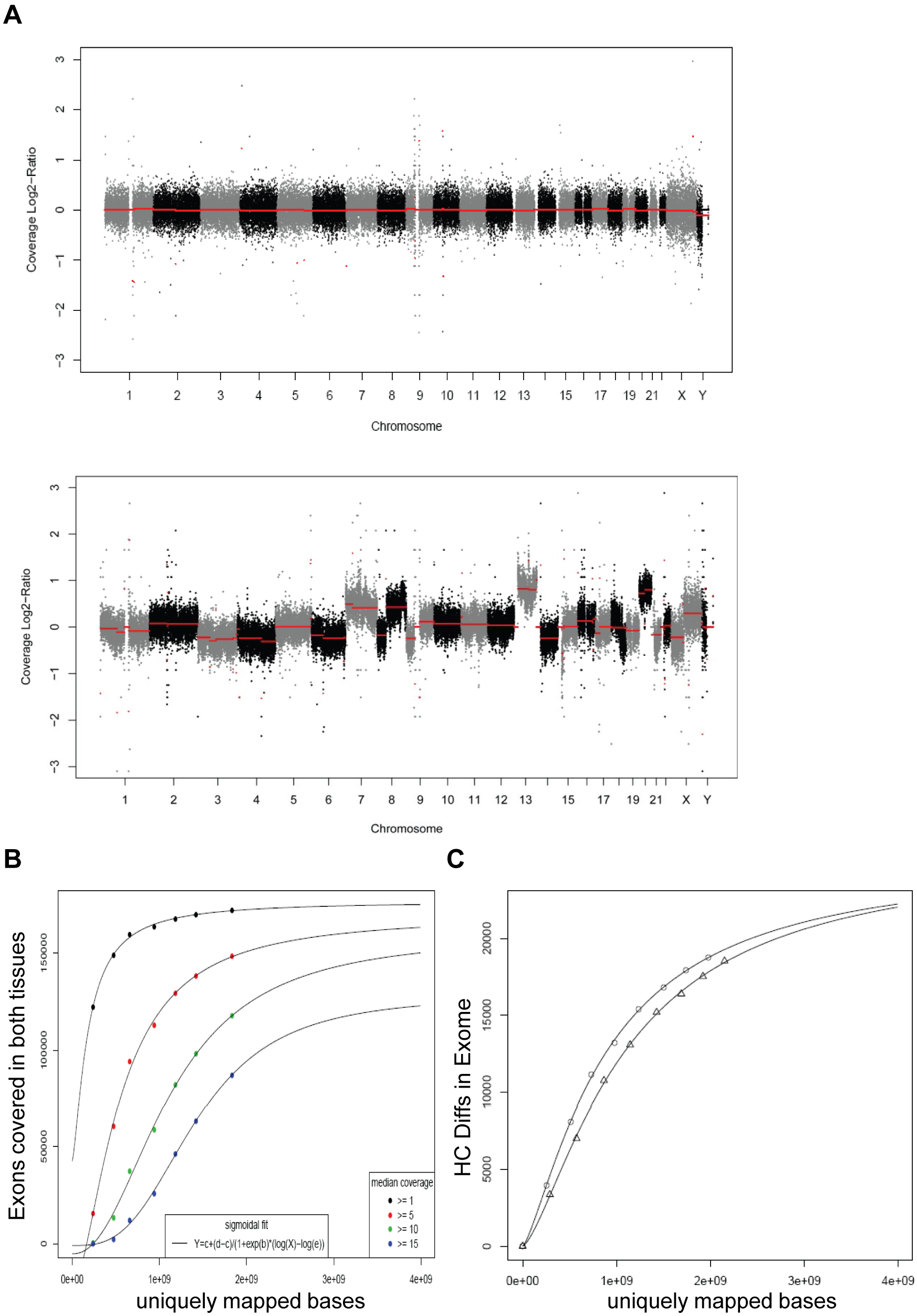

Supplement: Figure S1 — Quality controls of the colon cancer case 1 and experimental performances. (A) Visualization of Copy number variations (CNV) using Illumina sequencing for MSI and MSS cancers. Chromosomal coverage ratio of tumor versus benign tissue sample. Each chromosome was divided into 50-kb bins. The log2 ratios of unique reads per bin are plotted across all chromosomes. The red lines depict the local averages as calculated by DNAcopy [35]. (B) Influence of sequencing depth on exon capture coverage (left) and SNV detection (right). Exon coverage and SNVs in the enrichment regions were determined after each sequencing run. The numbers of exons covered and the number of SNVs detected at different coverage levels were compared for tumor and benign tissue separately. Sigmoid functions Y = c+(d-c)/(1+exp(b)*(log(X)-log(e)) were used to fit the data and extrapolate the saturation level. (TIF) [file pone.0015661.s001.tif]

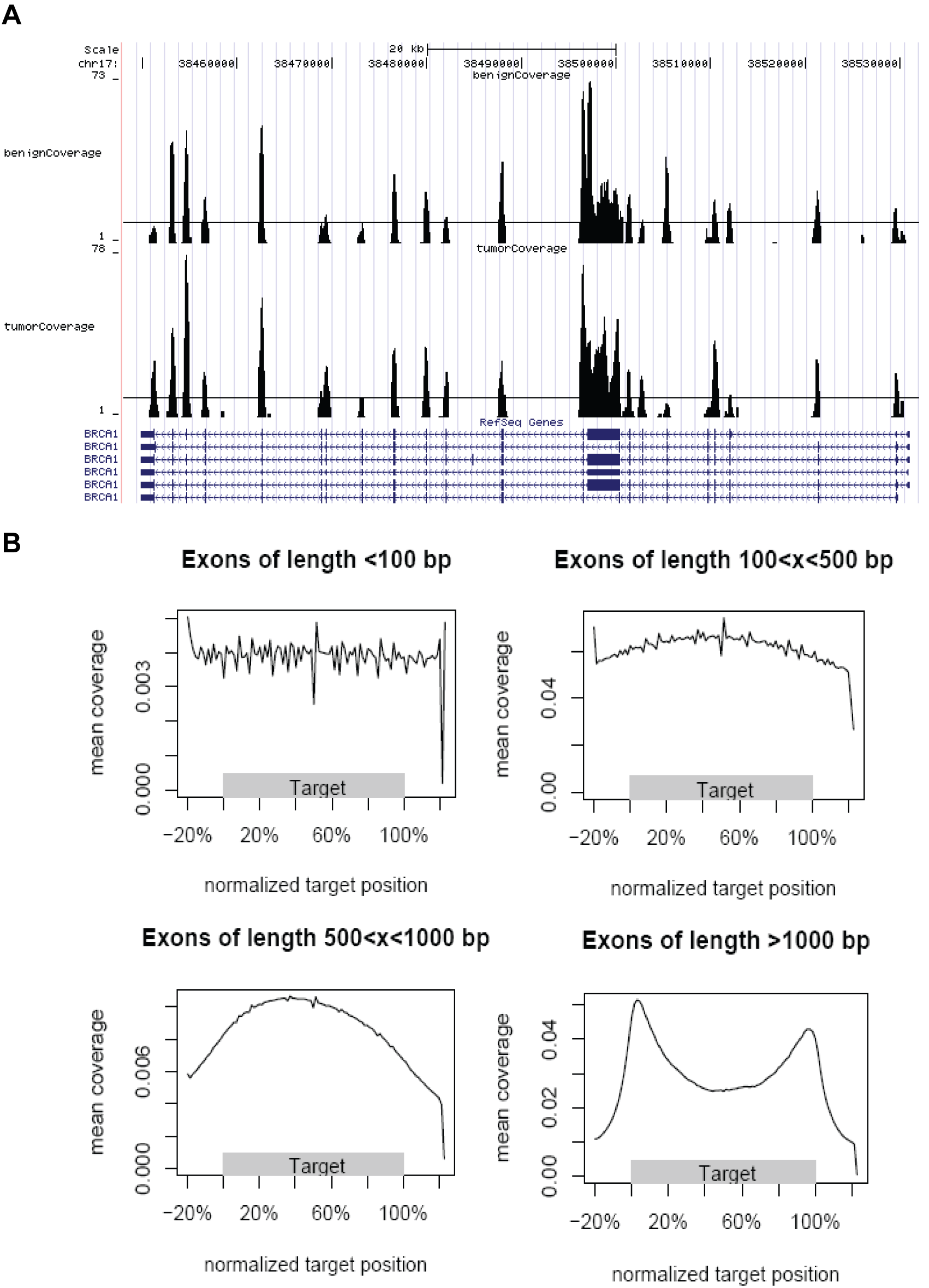

Supplement: Figure S2 — Sequence coverage along a contiguous target. (A) The base-by-base sequence coverage along a typical 80-kb segment (BRCA1 gene) in the UCSC browser is shown. The 10-fold coverage level is highlighted by a black line. (B) Coverage profiles of exon targets depending on exon size. Exons have been divided into four groups depending on exon size. Coverages were calculated in relation to the relative position on the exon and averaged by the mean over all exons of the group. (TIF) [file pone.0015661.s002.tif]

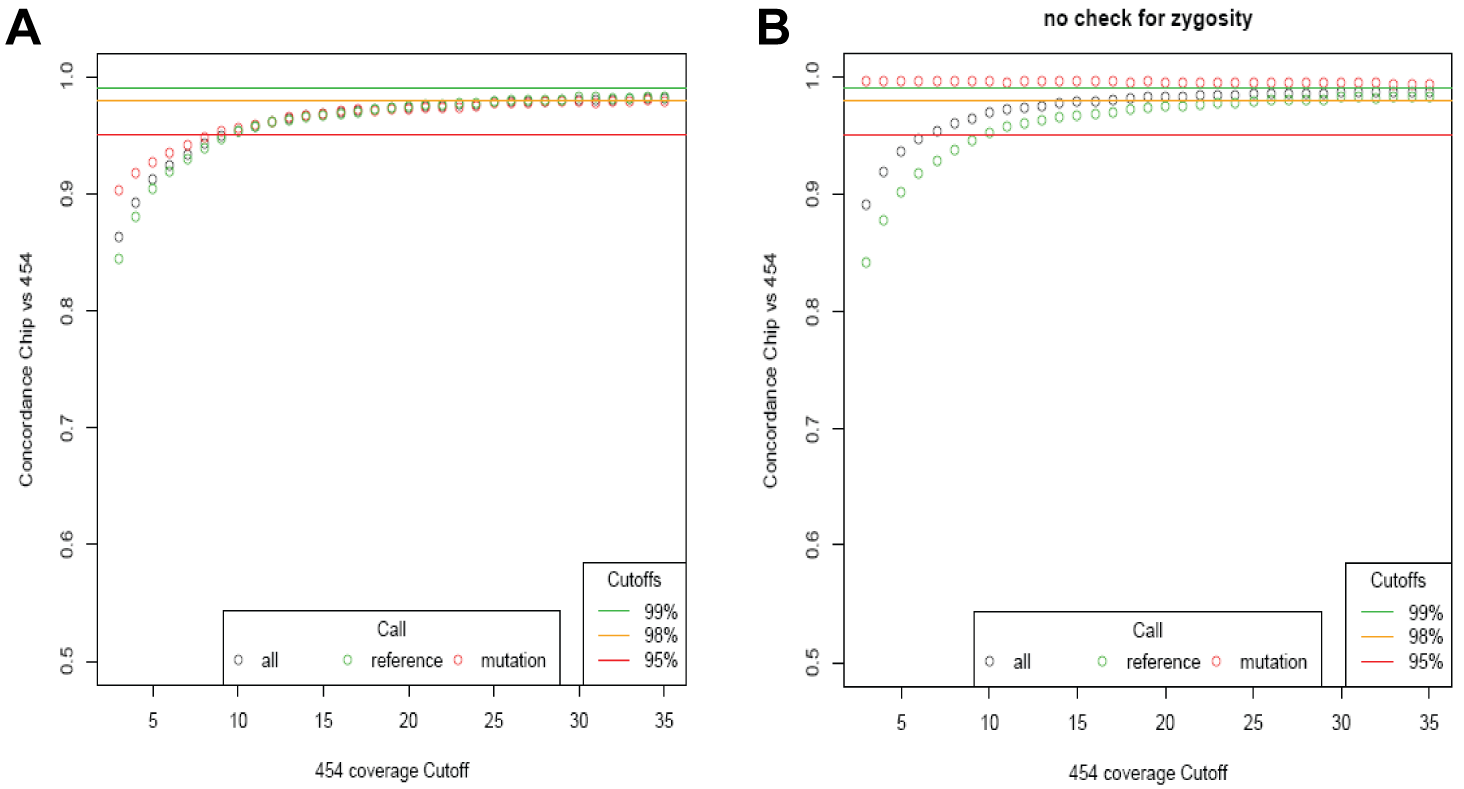

Supplement: Figure S3 — Comparison between the SNP array and NGS. About one million known SNP positions have been investigated using the Affymetrix human whole genome SNP array 6.0. Array positions with a quality score (p-value)<0.1 and sequencing positions with coverage exceeding 3-fold coverage were used for comparison. Forty thousand and thirty-six thousand positions for tumor and benign tissue, respectively, were eligible for comparison. To determine false positive and false negative rates, the array data was set as standard and between reference call and SNP call dependence on the array data was distinguished. (A) homo- and heterozygous SNVs were discerned (B) for the calculation of the haploid concordances heterozygous positions were counted as homozygous non-reference positions. (TIF) [file pone.0015661.s003.tif]

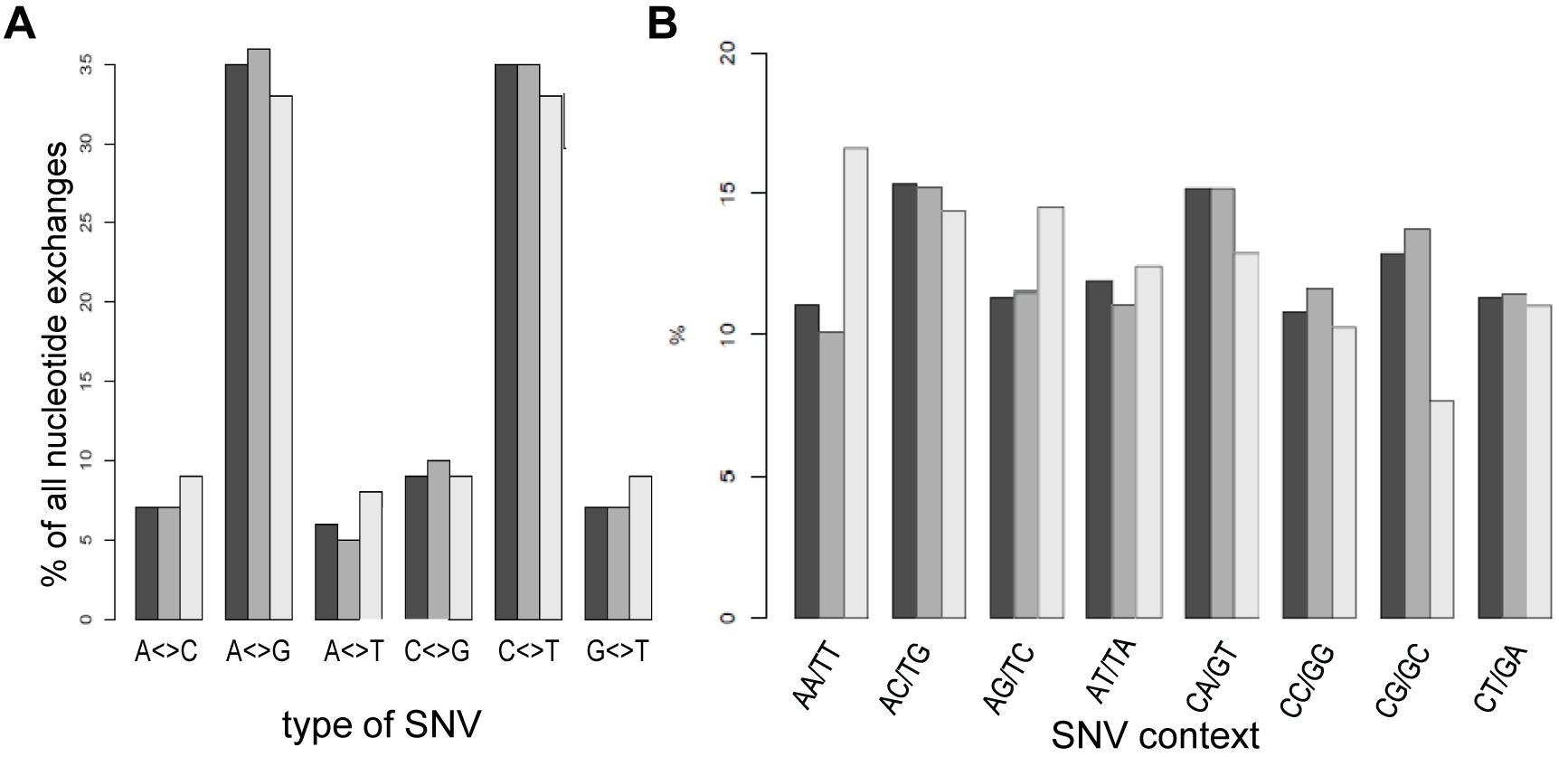

Supplement: Figure S4 — Nucleotide exchange rates in DNA from tumor and benign tissue, as compared to dbSNP130 data for patient 1. (A) Using the GS Reference Mapper Version 2.0.0.12 (Roche), software nucleotide exchanges were calculated for all possible transitions (e.g. A<>G, A<>C). Dark grey: tumor, grey: benign, light grey: dbSNP (B) Dinucleotide context for single nucleotide variants from tumor and benign tissue. Dark grey: tumor, grey: benign, light grey: dbSNP (TIF) [file pone.0015661.s004.tif]

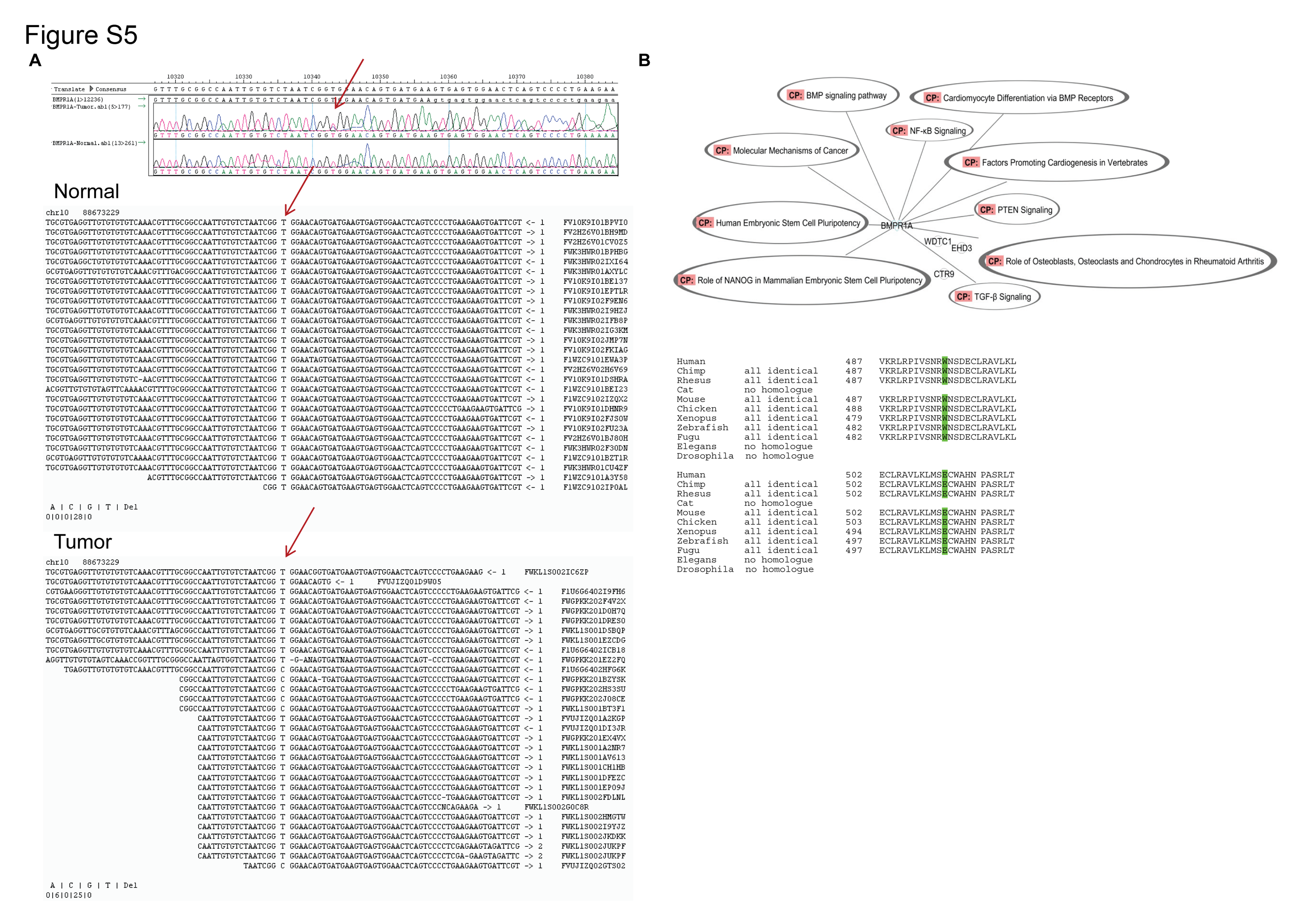

Supplement: Figure S5 — Validation, visualization and pathway analyses. (A) Visualization of the Sanger and 454 next generation sequencing result of BMPR1A. Red arrows indicate the location of the mutation. (B) Ingenuity pathway analysis of BMPR1A, WDTC1, EHD3 and CTR9 (top) and visualization of the conservation of BMPR1A p.W487 and p.E502 across human, mouse, chicken, zebrafish and other organisms (bottom). (TIF) [file pone.0015661.s005.tif]
